# Salmon louse (*Lepeophtheirus salmonis*) transcriptomes during post molting maturation and egg production, revealed using EST-sequencing and microarray analysis

**DOI:** 10.1186/1471-2164-9-126

**Published:** 2008-03-10

**Authors:** Christiane Eichner, Petter Frost, Bjarte Dysvik, Inge Jonassen, Bjørn Kristiansen, Frank Nilsen

**Affiliations:** 1 Institute of Marine Research, Bergen, Norway; 2 Department of Informatics, University of Bergen, Bergen, Norway; 3 The Norwegian microarray consortium, Institute for molecular bioscience, University of Oslo and The Norwegian Radium Hospital, Norway; 4 Computational Biology Unit, Bergen Center for Computational Science, University of Bergen, Norway; 5 Current address: Intervet Norbio AS, Thormohlensgt. 55, N-5008 Bergen, Norway; 6 Current address : Department of Biology, University of Bergen, Norway

## Abstract

**Background:**

*Lepeophtheirus salmonis *is an ectoparasitic copepod feeding on skin, mucus and blood from salmonid hosts. Initial analysis of EST sequences from pre adult and adult stages of *L. salmonis *revealed a large proportion of novel transcripts. In order to link unknown transcripts to biological functions we have combined EST sequencing and microarray analysis to characterize female salmon louse transcriptomes during post molting maturation and egg production.

**Results:**

EST sequence analysis shows that 43% of the ESTs have no significant hits in GenBank. Sequenced ESTs assembled into 556 contigs and 1614 singletons and whenever homologous genes were identified no clear correlation with homologous genes from any specific animal group was evident. Sequence comparison of 27 *L. salmonis *proteins with homologous proteins in humans, zebrafish, insects and crustaceans revealed an almost identical sequence identity with all species.

Microarray analysis of maturing female adult salmon lice revealed two major transcription patterns; up-regulation during the final molting followed by down regulation and female specific up regulation during post molting growth and egg production. For a third minor group of ESTs transcription decreased during molting from pre-adult II to immature adults. Genes regulated during molting typically gave hits with cuticula proteins whilst transcripts up regulated during post molting growth were female specific, including two vitellogenins.

**Conclusion:**

The copepod *L.salmonis *contains high a level of novel genes. Among analyzed *L.salmonis *proteins, sequence identities with homologous proteins in crustaceans are no higher than to homologous proteins in humans. Three distinct processes, molting, post molting growth and egg production correlate with transcriptional regulation of three groups of transcripts; two including genes related to growth, one including genes related to egg production. The function of the regulated transcripts is discussed in relation to post molting morphological changes in adult female salmon louse. There is clear evidence that transcription of the major yolk proteins is not induced before some of the post molting growth of abdomen and the genital segment has occurred. A hallmark for the observed growth is transcription of many putative cuticula proteins prior to the size increase.

## Background

Copepods are arthropods in the aquatic environment and the extremely abundant free-living species are an essential part of the first levels of the marine food chain. Although copepods comprise the largest animal biomass on the earth, relatively limited biological information is available at the molecular level and no model species exists.

The salmon louse (*L. salmonis*) is an ectoparasitic copepod feeding on skin, mucous and blood from salmonid hosts. Recently it was shown that *L. salmonis *infections in farmed fish induce epizootics in wild fish [1,2]. The life cycle of *L.salmonis *consists of 10 developmental stages separated by ecdysis [3,4] and after the final molt, females develop into mature adults that continuously produce eggs for life. The first free-living larvae (naupli I) hatch directly from egg-strings attached to adult females and all three larval stages (naupli I, naupli II and the infectious copepidid stage) can be transported by the ocean currents over large distances depending on hydrographical conditions [5]. After host settlement the infectious copepodids stage molt into chalimus. The four chalimus stages, all separated by molting, are anchored to the host by a frontal filament [6], which restricts the feeding area. However, in the succeeding pre-ad I and -II and adult stages the salmon louse can move unrestricted on the host surface resulting in increased virulence [7].

Sexual maturation and vitellogenesis are major physiological and behavioral changes in most animal life cycles. Germ cells are typically established early in development but arrested in development until the onset of sexual maturation. The generation of gametes is most conserved in males whereas variation is seen between different species for the development of female gametes. The ovum (i.e. the mature unfertilized egg) is a highly complex cell that is energetically expensive to produce. In order to produce high quality ova the females must undergo physiological adaptations that initiate further gamete development and maturation. Since the reproduction strategy is highly variable between different species and different life strategies (e.g. free living or parasitic) the processes of sexual maturation and the production of eggs also varies. However, there are some common hallmarks that are expected in most animals. After fertilization, the egg must contain sufficient energy to ensure development until external energy sources can be utilized. The ovarium is the site of initial development for female gametes during sexual maturation and reproduction. At ovulation, oogonia are released from the ovarium into the oviduct where growth and maturation take place. This process is typically divided into pre-vitellogenic and vitellogenic development. During vitellogenesis, yolk proteins are incorporated into the oocytes. A molecular hallmark for this process is the transcription of genes encoding egg yolk proteins like vitellogenins (Vgs). Depending on animal group, the transcription of Vgs takes place in different cell types like liver (in vertebrates), fat body (in insects) and hepatopancrease (e.g. in decapods). The Vgs are subsequently transported by the blood or hemolymph to the maturing oocytes, where they are taken up by receptor-mediated endocytosis [8,9]. Production of vitellogenin is controlled by steroid hormones, which induce transcription of the target gene through binding of a steroid-receptor complex to the gene promoter. In arthropods, including crustaceans, ecdyson (i.e. E20) has been shown to bind to the heterodimeric ultra-spinacle (UsP) and ecdysteroid receptor (EcR) to an ecdysteroid response element (ERE) in the vitellogenin promoter [10]. Ecdysteroids are also a key regulatory component in arthropods molting and development [11,12].

The salmon louse reproductive systems have been described at the anatomical and histological levels [13] but there is no information regarding the timing of the different events during sexual maturation. It has been proposed that males depositing spermatophores triggers the egg-production in other parasitic copepods (*Lepeophtheirus pectoralis*) [14], but according to our observations using unfertilized laboratory animals, adult female *L. salmonis *produce eggs and external egg string also when males are not present (pers. obs., present study). Unlike crustaceans like shrimp, that produce eggs in seasons and grow/molt their entire life, salmon louse have a final molting, stop growing when egg production has started and then continuously produce eggs for life. It has been shown that *L. salmonis *can produce up to 11 sets of egg-strings from a single fertilization [15]. However, immediately following the last molting, the adult female salmon louse is not fully developed. Prior to egg production the animal matures in a process that includes a large increase of the genital segment and the abdomen, whereas the frontal cephalothorax appears unchanged (present study).

In order to link transcripts to the morphological and anatomical changes that takes place during the transition from pre-adult II to egg producing females we have combined EST sequencing and microarray analysis. The microarray analysis revealed three distinct groups of transcripts that correspond to molting, post molting growth and egg production. The possible function of the regulated transcripts is discussed in relation to the anatomical and physiological changes taking place. Initial analysis of EST sequences of *L. salmonis *revealed a large proportion of transcripts with no significant hits in public databases. In order to obtain some initial information regarding copepod proteomes we compared at set of *L. salmonis *proteins to some selected crustaceans, insects and vertebrates and shows that the salmon louse proteins are equally similar to all species.

## Results

### EST analyses

**Figure 1:**
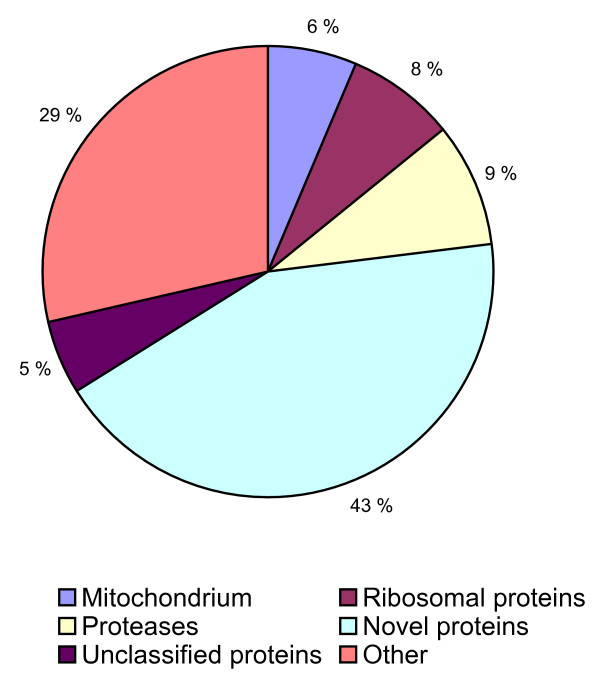
Distribution of individual ESTs in major functional groups based on primary annotation.

During the course of the present study 7,021 ESTs from five cDNA libraries (adult and preadult stages, un-normalized libraries) were sequenced (Table 1). The initial annotation of the individual ESTs revealed that 43% of the sequences had no significant hit in GenBank and 5% showed significant hits with proteins with unknown function (Figure 1). Nine percent of the ESTs gave hits with proteases, 8% with ribosomal proteins and 6% with mitochondrial transcripts, of which 81.6% were 16S rRNA [16]. The high number of novel transcripts in *L. salmonis *was also evident when we compared our ESTs against the recently available first crustacean genome of *Daphnia pulex *[17]. Compared to the *D. pulex *proteins, 51% of our validated nuclear *L.salmonis *ESTs (4,563 ESTs) gave non-significant hit. Assembly of all non-mitochondrial sequences with a length over 100 bp (4,563 ESTs) resulted in 556 contigs (2,948 ESTs) and 1614 singletons (Table 2). The majority of the 556 contigs contained 2–4 ESTs and initially only two clusters contained more than 100 EST. Contig63 contained 147 ESTs encoding trypsin (*LsTryp1 *and *LsTryp2*) transcripts [18-20], while Contig72 (124 ESTs) encodes a novel gene with no detectable conserved domains in the putative protein (178aa) encoded by the open reading frame (ORF).

**Table 1:** Overview of isolated clones and sequenced ESTs from the various cDNA libraries

Library	N clones	Percent of total	N EST*	Percent of total
Female total	4,320	61.5	3,262	63.8
Female intestine	864	12.3	464	9.1
Adult male	768	10.9	646	12.6
Pre-adult II female	768	10.9	571	11.2
Female male subtracted	301	4.3	166	3.2

Total	7.021	100.0	5.109	100.0

* Sequences with a remaining length over 100 after vector trimming and quality assessment.

**Table 2:** Summary of data obtained after EST assembly using the ContigExpress module in Vector NTI.

	ContigExpress analysis
Sequences analysed:	4563
Number of ESTs in contigs:	2948
Number of contigs:	556
Number of singletons:	1614
Number of contigs containing:	
2–4 ESTs	418
5–10 ESTs	92
11–20 ESTs	25
> 20 ESTs	16

About 1,900 clones were not included in the contig assembly, primarily due to lack of high quality sequence data but also due to insert sizes less than 100 bp (< 1%) or empty clones/*E. coli *sequences (1%). Based on these considerations, the proportion of singletons (35%) in the clustering results and the proportion of mitochondrial sequences (6%), the number of additional salmon louse transcripts among the 1,900 clones without sequence data were estimated to be approximately 500. This indicates that the 7,021 clones, from which cDNA probes were printed on the microarray, represent up to 2,600 different *L. salmonis *transcripts.

The initial annotation (see Methods) showed no clear high correlation with homologous genes from any specific animals of animal groups, for instance other arthropods. In fact sequence comparison of 27 *L. salmonis *proteins with homologous proteins in humans, insects (*Aedes aegypti, Drosophila melanogaster, Anopheles gambiae*), crustaceans (see Methods) and zebrafish (*Danio rerio*) revealed an almost identical sequence identity (range: 26–100%, mean: 65.5%) for all 27 genes independent of whether the *L. salmonis *sequences were compared with terrestrial arthropods, crustacean, fish or humans (Table 3).

**Table 3:** Sequence identity analyses of *L.salmonis *proteins with homologous proteins in crustaceans, insects, fish and humans

**Contig no.**	**Annotation**	**No AA**	**Crustaceans**	***Aedes aegypti***	***Drosophila melanogaster***	***Anopheles gambiae ***	***Danio rerio***	***Homo sapiens***
Contig 504	Actin	376	95	96	96	96	95	95
Contig 15	40S ribosomal protein S13	151	85	80	79	80	84	87
Contig 20	60S ribosomal protein L5	296	67	68	67	66	66	68
Contig 211	Glyceraldehyde-3-phosphate dehydrogenase	332	76	74	74	76	76	75
Contig 222	QM protein	219	81	78	81	79	77	77
Contig 233	60S ribosomal protein L7A	266	69	66	64	60	68	68
Contig 286	translation initiation factor 4A2 isoform 2	413	71	69	69	69	72	73
Contig 307	glucosamine-6-phosphate isomerase	268	70	72	71	71	69	70
Contig 340	elongation factor 1-alpha	459	71	76	77	76	74	74
Contig 392	receptor for activated protein kinase C-like	316	85	79	80	78	78	78
Contig 468	14-3-3-like protein (Leonardo protein)	252	80	83	85	82	81	79
Contig 73	Adenosylhomocysteinase	411	76	73	73	73	73	73
Contig 81	S5e ribosomal protein	213	84	83	81	77	89	84
Contig 83b	phospholipid-hydroperoxide glutathione peroxidase	182	65	54	42	46	59	52
Contig 109	Adenosine kinase (AK)	332	38	50	45	44	50	51
Contig 192	Calmodulin (CaM)	149	100	100	100	100	97	97
Contig 229	Cystathionine-beta-synthase	381	58	54	52	52	59	57
Contig 264	histone H4	103	100	100	100	100	99	99
Contig 325	ER protein disulfide isomerase	401	56	55	53	53	42	52
Contig 363	Enolase	290	76	76	76	76	74	74
Contig 28	serine-type enodpeptidase (trypsin-lik)	226	38	37	34	35	32	28
Contig 29	metalloproteinase	322	35	38	36	38	37	31
Contig 56	serine protease	254	31	31	30	34	29	25
Contig 88	cathepsin L	312	54	51	51	50	50	49
Contig 193	cysteine protease (cathepsin)	372	31	31	32	31	33	33
Contig 434	carboxypeptidase	241	40	29	33	30	26	27
Contig 131	heat shock protein 70	335	69	76	75	76	79	78

**Mean**			**66.7**	**65.9**	**65.0**	**64.7**	**65.5**	**65.0**

### Post-molting growth of adult female *L.salmonis*

**Figure 2:**
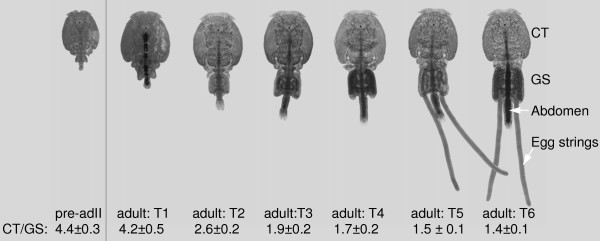
**Post molting growth of adult female *L. salmonis***. The size of the cephalothorax (CT) is stable, while the genital segment (GS) and the abdomen (Ab) grow as indicated by the decreasing CT/GS ratio. The time frame from T1–T6 is approximately two weeks at 10°C. External egg strings may be observed approximately 10 days post molting.

The salmon louse is characterized by four main body parts. After the final molt into adult females, the genital segment and the posterior abdomen showed a substantial growth, whereas the large anterior cephalothorax and the small free thoracic segment remained constant (Figure 2). At 10°C the metamorphosis from T1–T6 was finalized in approximately 13–18 days and adult female T6 lice continued to produce a new set of egg strings approximately every 10 days. The growth correlated with the presence of maturing egg strings within the genital segment. Maturing oocytes were observed at T2 and the presence of ova evident at T4. After the mature egg-strings were extruded at T5, two new immature egg-strings entered the "empty" genital segment. Between each time point external egg-strings were extruded as new eggs continuously matured within the genital segment.

### Microarray analysis

Microarray analysis was performed on a total of 34 female *L. salmonis*, representing the pre-ad II stage and 6 different time points after the last molting (Figure 2). ESTs were grouped according to expression profiles using self-organizing map (SOM) [21,22] (25 cluster (5 × 5) neuron map). The SOM revealed three clusters with a substantial increase in gene transcription during adult development (clusters 1, 2 and 6) and two (clusters 24 and 25) with a significant decrease (see Additional file 1). All other SOM clusters showed very little variance within clusters. Although the groups sampled are not clear-bordered developmental stages but rather transitions in time, correspondence analysis (CA) [23] of the complete dataset (see Additional file 2) revealed consistency among the 5 biological parallels selected according to the genital segment/cephalothorax ratio (see Figure 2). With the exception of one obvious pre-ad II outlier, samples were located in adjacency with other samples from the same group or in the vicinity of a neighboring time point. The developmental group membership, and the single outlier, of individual samples were also evident in a 10 × 10 neuron heat SOM (see Additional file 3).

**Figure 3:**
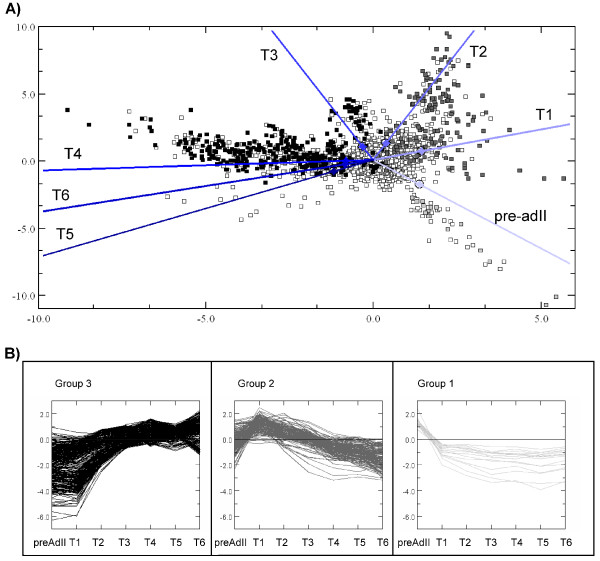
**Correspondence analysis plot (A) and expression profiles of identified gene sets (B)**. Spots represent individual EST and vectors represents sample group trimmed means. Three groups of annotated genes after quality check are shown, representing the group 1 ("late genes") up-regulated from T2 (black spots or profiles respectively), the group 2 ("early genes") up regulated at T1 and subsequently down regulated (dark grey spots or profiles respectively) and the group 3 ("immediately early") genes up regulated in pre-ad II and subsequently down regulated (light grey spots or profiles respectively). Any gene located near the plot origin is poorly correlated with any of the principal axes and sampled groups. Correlation to both the principal axes and sample groups increases as genes are located further from the origin in the direction of one of the axes or sample group vectors.

Correspondence analysis using trimmed mean values for all lice from each time point, reducing the influence of outliers, shows that the main direction genes are connected with the respective developmental time point (Figure 3). The two first principal axes explain 89% of all variation in the data set. The vectors corresponding to time point's fall into an anti clockwise pattern for the sequential transition during time and, as expected, the vectors representing the most different developmental stages are on opposite sides in the plot. Most microarray probes show little variation in gene expression over the time points measured. These ESTs are found in the centre of the CA plot while differentially expressed ESTs that are clearly connected with developmental stages are located far from the centre and along one of the development stage vectors. Two major and one minor cloud of ESTs, strongly separated from the centre, are evident in Figure 3. The two main patterns; 1) up-regulated from T2 to T6 and 2) up-regulated from pre-ad II to T1 followed by down regulation from T1 to T6, accounts for 14% of all ESTs and are almost completely correlated to the first principal component analysis. A third minor group of ESTs strongly correlates with the pre-ad II group and consists of ESTs transcribed in the pre-ad II stage but down regulated in adult T1.

**Figure 4:**
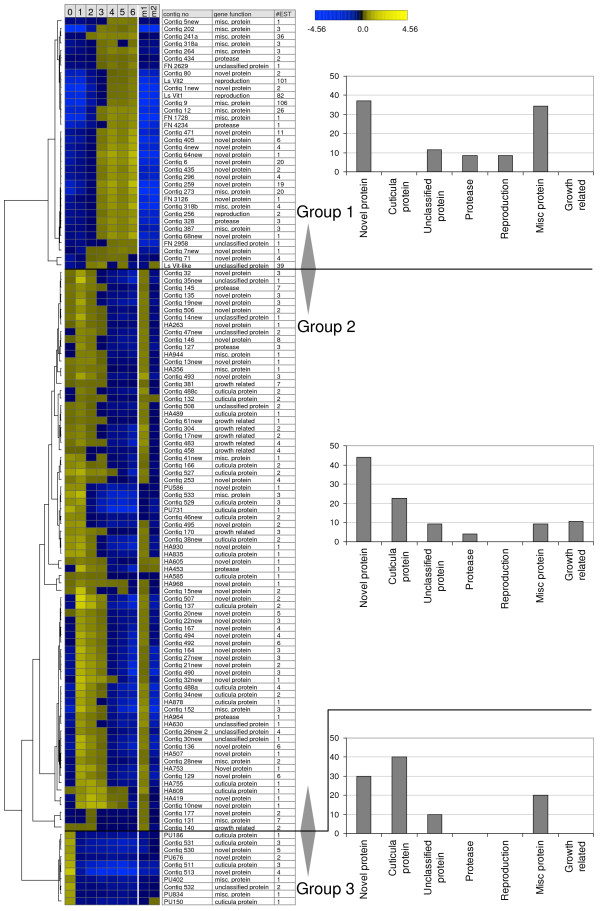
**Hierarchical clustering of merged contigs**. The columns of the heat map represents mean group signal (mean EST log, sample vs. reference). Column 0 represents pre-adII females, columns 1–6 represents adult female post molting growth time points T1–T6, respectively (according to Fig 2) and columns m1 and m2 represents male groups, preadII and adults, respectively. Three distinct patterns can be seen for differentially transcribed genes in adult females; the upper sub-tree shows genes up-regulated from T2 and during most of the maturation phase (group 1). The middle sub-tree shows genes that are turned on in T1 and but down-regulated during the post molting growth (group 2). The lower sub-tree shows genes transcribed in pre-ad II but down regulated throughout post molting growth in adult phases of the experiment. Male, pre-adII and adult transcription levels of genes regulated during female molting and post molting growth are shown in the right section of the dendrogram (m1 and m2) for sex comparison.

Group 1 (up-regulated from T2 to T6) defined from the 5 × 5 SOM, contain 573 ESTs (cluster 1, 2 and 6), group 2 (up-regulated from pre-ad II to T1 followed by down regulation from T2 to T6) contains 267 ESTs (cluster 24 and 25) while group 3 (down regulated from pre-ad II to T1) defined from the CA contains 49 ESTs. The data underlying the corresponding expression profiles were re-examined for quality (probe size, double-probes, signal to noise ratio) and 414 ESTs were kept (277 up, 137 down). Only one of the remaining ESTs encodes a housekeeping gene that could be expected to be unregulated (*RPL19*). Since the array contains a high number of 16S rRNA mt probes and ESTs encoding ribosomal proteins, this demonstrates a low number of false positives hybridizations. In addition, within contigs 98.3% of the individual ESTs encoding the same gene had the same transcription profiles. Due to this low error rate quality-checked singletons were also included in the further analysis. All the final contigs and singletons were *in silico *re-annotated after manually inspecting and editing the contig sequences, and after re-sequencing the singletons using both vector primers. ORFs larger than 130aa were used in BlastP search in Genbank against the non-redundant (nr) database and the entire cDNA sequences were used in translated (BlastX) search. The highest scoring sequences were used to indicate the putative function with emphasis on identifying groups of biological function. After the sequence re-examination, group 1 and 2 contained a total of 73 contigs and 37 singletons while group 3 contained 6 contigs and 4 singletons but the numbers of novel genes were still high in all groups 1–3, 38%, 43% and 30%, respectively (Figure 4). Various functions were suggested for the non-novel genes (see Additional file 4 for the complete list). Some of the annotation results may be biased by the fact that out of the 120 contigs/singletons showing significant regulation in the microarray analysis, 25 had two or more overlapping ORFs larger than 130aa, and several of the alternative ORFs gave significant hits in BlastP searches. In fact multiple reading frames were evident in many full-length *L. salmonis *transcripts. Out of the 27 full-length transcripts used for comparison with homologous genes in other species, 5 (18,5%) had additional ORF encoding putative proteins over 130aa (See Additional file 4), including ORF with highly significant annotation hits. For example, on the transcript encoding *L.salmonis *Actin (Contig 504), 376aa ORF on the poly A strand (+1 frame), a 277aa ORF on the negative strand (-1 frame) encodes a putative protein highly similar (E-value 2E -41) to a hypothetical protein in Chimpanzee and a putative mRNA splicing factor in *Nasonia vitripennis *(E-value 2E-36). The hypothetical chimpanzee protein is on chromosome 14 and not on the same chromosome as chimpanzee actin-like genes.

The majority of the annotated group 2 and 3 "early genes" (T1 or pre-ad II transcribed) were similar to cuticula proteins (22 and 40% respectively), especially Barnacle cypris larva-specific gene (*BCS-1*) from *Balanus amphitrite *[24], a marine crustacean, but the E-values were high (see Additional file 4). In addition, 8% of the group 2 genes were similar to genes involved in other growth related processes. Most group 2 contigs were also down regulated in adult male lice compared to pre-adII males (Figure 4), pointing towards sex independent functions. However, in group 3 (down regulated in females from pre-ad II to adult T1) all but one gene was unregulated in males.

All the salmon louse contigs homologous to cuticula-like proteins were compared to each other and based on the size of the encoded protein they separated into two sub-groups. These sub-groups could be divided into several other groups based on sequence similarity. Some short proteins (HA608, HA755, Contig137, Contig511, PU731 and PU186) are similar to *BCS-1 *while other cuticula proteins (Contig488, Contig34new, HA878) are similar to cuticula protein 6 in insects. Aligning these sequences to a selection of sequences from insects revealed some degree of conservation (a few stretches of conserved amino acids) indicating similar functions. The Contig166 and Contig527, similar to horseshoe crab (*Tachypleus tridentatus*) cuticular protein have a conserved domain, a chitin binding Peritrophin-A domain (pfam 01607.11) found in chitin binding proteins in the perotrophic matrix proteins of insects [25-27]. Interestingly, a perotrophic membrane is not described in *L. salmonis*.

The highest scoring sequences among the "late genes", up-regulated during adult female maturation, gave hits with genes involved in reproduction. Two large contigs (Contig552 and Contig276) showed high level of identity with two different arthropod vitellogenins [28,29] and Contig256 had similarities to a nucleolar protein in starfish (*Asterina pectinifera*) only transcribed in growing oocytes [30]. Furthermore, except for one gene (*LsVit-like*) all group 1 genes were up regulated in females only (Figure 4).

None of the 86 "early genes" (group 2 and 3) were in contigs consisting of more than 10 ESTs compared to 10 of the 34 "late genes" (Figure 4), including 3 genes encoding novel proteins (Contig471, Contig259 and Contig6). Five "late genes" (Contig12, Contig9, Contig241, Contig273 and *LsVit-like*), within the group of genes with miscellaneous functions, were also in large contigs (20–106 ESTs) indicating a high transcription level. The Contig9, that contains more ESTs (106) than both vitellogenins, gave strong hits with a Cathepsin L-associated protein in *Artemia *[31] mainly because it contains three FAS1 domains typically seen in cell adhesion molecules [32,33].

The microarray transcription profiles were confirmed by Northern blot analysis using 2 genes in Group 1 (Contig387, Contig12) and 5 genes in Group 2 (Contig529, Contig488a, Contig34new, Contig533, Contig508) (See Additional file 5).

## Discussion

Salmon lice included in the present microarray study are female pre-adult II (before the final molt) and adults at different time points (T1–T6) during the post molting growth towards egg-production (see Figure 2). The microarray analysis of approximately 2,600 different transcripts revealed a total of 120 regulated genes divided into 3 patterns, down regulated from pre-ad II to adult T1 (group 3), up-regulated from pre-ad II to T1 and then down regulated (group 2), and up-regulated from T2-T6 (group 1). A typical feature of group 2 and 3 is transcripts resembling cuticula proteins, while the group 1 typically includes female specific transcripts, including vitellogenins, with many ESTs (up to 106) compared to the contigs in group 2 and 3. The diversity of transcripts is largest in group 2.

### Cuticula encoding genes are transcribed prior to the final molt and prior to the post molting growth of adult female salmon louse

Animals with an exoskeleton, like the salmon louse, grow through molting and each instar typically shows limited increase in size. Developmental stages in salmon louse (3 free-living and 7 parasitic) are also separated by molting [34]. However, the present study clearly demonstrates a substantial growth in females after the final molt. Post molt growth has been described in another parasitic copepod, *Lernaeocera branchialis*, where substantial growth and metamorphosis in adult females after the final molt have been reported, resulting in a 20-fold size increase of the abdomen [35]. They observed extensive cuticular folds (4–6 um deep folds, with a density of 1–1.2 folds/um), associated with expansion of the cuticle following the final molt, which could account for some of the size increase (about 6-fold). Based on ultra structural observation they concluded that additional mechanisms (i.e. other than stretching of the cuticular folds), like large-scale cuticule secretion must account for the large size increase. In *L. salmonis *small folds (0.9 um) have been observed in the cuticula of chalimus larva [36], but currently there is no detailed description of the development of salmon louse after the final molt. Our preliminary observations on T1 and T6 lice indicate clear differences in cuticula structure, particularly on the genital segment and abdomen. Immature adult *L. salmonis *(T1) has cuticular folds but to what extent these folds can explain the large increases in size during the post-molt growth observed in the present study is not clear. Prior to the final molting (pre-ad II lice) and at the start of the post molting growth (T1) an increase in transcription of genes believed to be involved in growth related process in arthropods (e.g. cuticula proteins) was observed. During the post molting growth these transcripts were down regulated while female specific transcripts were up regulated. Egg strings, from which the offspring (planktonic nauphli larvae) is released, were not produced until this post molting growth was completed. This indicates that the post molting growth is connected to sexual maturation for egg production (increased genital segment) and an increased capacity for food (mainly blood) uptake (increased abdomen). The high proportion of cuticula and growth related genes identified in group-2 and -3 (25 and 40%, respectively) indicates that the group-2 and 3 genes, including the novel proteins (43% and 30%), primarily consists of genes involved in copepod growth. Many of the pre-ad II and T1 transcribed cuticula annotated genes (group 3 and 2, respectively) resemble *BCS-1*, known to be transcribed prior to Barnacle larval attachment and metamorphosis [24]. For pre-ad II lice this could be related to the fact that the louse is attached to the salmon host through a frontal filament during molting. However, this is somewhat contradicted by the fact that the pre-ad II transcribed *BCS-1 *like genes are not regulated in males. Since both group 3 and 2 genes are down regulated after T1, when the post-molting growth of adult female salmon louse takes place (see Figure 2), growth from T1 to T6 is phenotypically delayed in time relative to transcription and probably do not include cell division growth. This is consistent with the fact that the new cuticula is produced beneath the old pre-ad II cuticula, prior to the molting when the group 3 genes are transcribed. Therefore in order to increase in size some kind of stretching/swelling must occur after the old cuticula has been shed, starting at T1 when the group 2 genes are transcribed. Based on the present study it is therefore likely that the group 2 and 3 cuticula proteins are an important part of a two-step process of cuticula formation at the final molt of *L. salmonis*.

### Abdominal growth and gene regulation

After the final molt, the *L. salmonis *abdomen grows from approximately 0.8 mm to 2.8 mm (3.5 fold length increase). It is likely that this increases the capacity for food digestion, which could be visualized as increased transcriptions of digestive peptidases. The *L. salmonis *microarray contains more than 600 peptidase ESTs but only seven of the regulated genes (three in group 1 and four in group 2) were identified as proteases (one trypsin-like, one cysteine peptidase, two carboxypeptidases and three metallopeptidases). None of the putative digestive serine peptidases previously identified in the salmon louse [18,19] were among the regulated transcripts. However, trypsin-like peptidases likely to be involved in digestion may also be regulated at the post-transcriptional level [19], and hence would not be detected in a transcriptome analysis.

Peptidases from the different families may be involved in a wide range of cellular and biological processes making it difficult to infer the function based on database searches. Host blood is a major component of the food for the salmon louse [37] particular for adult females. A wide range of organisms (both unicellular and metazoan species) utilize blood as nutrition. To keep blood in an easy accessible form metazoan species typically produce components with anticoagulation properties. In addition, it has been shown for several hematophagus organisms that digestion of blood and particular haemoglobin demands the action of a set of peptidases [38,39]. It appears that aspartic- cysteine- and metallo-peptidases are key players in blood and haemoglobin digestion from different parasitic species [38]. Of the regulated peptidases in the present study three are metallopetidases and one is cysteine peptidase. Contig127 (in group 2) is a cysteine peptidase resembling the trophozoite cysteine proteinase (TCP) from malaria (*Plasmodium falciparum*) [40]. Interestingly, the TCP is believed to be involved in degradation of erythrocyte haemoglobin, which also could be the case in the salmon louse as they ingest blood from their salmon host. In addition, Contig5new, which resembles a Kuniz-like serine peptidase inhibitor, is up-regulated from T1 to T6. Serine peptidase inhibitors are involved in a wide range of biological processes including blood coagulation/anticoagulation, hence, it is possible that Contig5new could encode an anticoagulant, which is up regulated due to the abdominal growth and the corresponding increase in blood feeding during egg production.

### Egg production and gene regulation

The female specific nature of all but one gene up-regulated during the T1–T6 growth of adult females (group 1) and the two identified vitellogenins, points towards genes involved in reproduction. No gene libraries were normalized and the high number of ESTs in many of the group 1 contigs, compared to group 2 and 3 contigs (Figure 4), therefore points towards functions where a high transcription level is typically seen. Both vitellogenin contigs (*LsVit1 *and *LsVit2*) contains approximately 100EST which is in compliance with the high transcription level typically seen for vitellogenins. The equally transcribed Contig9, encoding a protein containing 3 FAS1 domains, gave GenBank hits with CathepsinL-associated protein in *Artemia *where it is most abundantly expressed in encysted eggs and embryos [31]. It is therefore likely that Contig9 encodes a highly expressed protein with cell adhesion properties involved in egg production. In insects, FAS1 containing proteins are primarily described as neural cell adhesion molecules but they have also been reported to be present on the surface of eggs and also to be expressed in several non-neural tissues in the embryo [32,41]. Further information on the biological function of this gene and other genes in group 1–3 will be revealed through ongoing knock down studies.

Induction of the genes up-regulated during the post molting maturation of adult females appears to take place at time point T2 (see Figure 3). This is at the time when the first immature eggs are observed in the genital segment and 32% of the observed size increase has taken place. This, and the fact that similar growth appears in the abdomen, indicates that the final size of the genital segment in mature adults cannot be a result of stretching due to the presence of immature eggs only. From T3, the genital segment is gradually filled up with maturing eggs that are extruded as two separate strings (Figure 3). Based on these observations and the induction of a range of growth related genes (including cuticula proteins) at T1, it is likely that the observed size increase includes addition of more components into the exoskeleton rather than stretching.

### Hormonal regulation and transcription profiles in the salmon louse

In the present study salmon lice go through three distinct processes, molting from pre-ad II to adult, post molting growth and egg production, three processes that correlates with regulation of the three groups of transcripts (group 3, 2 and 1, respectively). In arthropods, the ecdysteroid 20-hydroxyecdysone (20E) and juvenile hormone (JH) are key participants in regulating growth (molting), sexual maturation and egg production [12,42]. In hematophagous mosquito a blood meal trigger the vitellogenesis, which is characterized by a specific gene expression pattern tightly controlled by the 20E [43]. In *Drosophila *short day photoperiods at low temperatures induce ovarian diapause and suppressed ecdysteroid production [44]. Temperature upshift results in an increase of steroid hormone levels, diapause termination and onset of vitellogenesis. Currently there is no available data about ecdysteroid levels in *L. salmonis*. In crustaceans (e.g. crabs, lobsters) information is also limited but it has been demonstrated that ecdysteroids are involved in molting, reproduction and embryogenesis [45]. In addition, ecdysteroid levels in free living copepods are known to fluctuate during the life cycle [46]. A recent microarray study in *Drosophila *assesed the genomic response of 20-hydroxyecdysone (20E) during metamorphosis [47]. By using RNAi to knock down EcR they were able to identify EcR-dependent genes. A total of 4,188 genes were regulated in EcRi animals at one or more of the examined times points, indicating that approximately 30% of the *Drosophila *transcripts are affected by EcR directly or indirectly. Dana et al. [48] examined ~2,000 transcripts related to blood feeding in *Anopheles gambiae *and found that approximately 20% of the examined genes were regulated as a response to the blood meal. These regulated transcripts could be separated into early, middle and late responding genes, similar to the present study, and were suggested to be controlled by the fluctuating titers of JH and 20E either as inducers and/or repressors of transcription.

The observed post-molt growth in *L. salmonis *is likely to be regulated at the transcription level since distinct peaks in transcription of cuticula and growth related genes is evident in pre-ad II, where new cuticula is produced, and in newly molted adult females prior to the post molting growth. Recently, it has also been shown that transcription of cuticular proteins can be induced by 20E in the beetle *Tenebrio monitor *[49]. Hence, it is likely that a 20E homolog is a key participant in inducing the transcription profiles observed in the present study, probably acting both as an inducer and repressor.

It has been proposed that fertilization is the trigger for the egg production (ecdysteriod inducer) in parasitic copepods [14]. However, at least in *L. salmonis *this is not the case since in the present study normal post molt growth, including production of external egg strings (with unfertilized eggs), was observed in female only populations. Furthermore, since egg producing *L. salmonis *females can be found on salmon throughout the year at all temperatures and light conditions, environmental factors do not seem to be a likely candidate. Although, egg producing females feed extensively on blood, and this high energy food is probably important for egg production, blood can be part of the diet at all parasitic stages indicating that vitellogenesis is not triggered by a blood meal either.

Recently it was shown that in *Drosophila *growth and sexual maturation is coupled, coordinated by the DHR4 nuclear receptor [50]. DHR4 is part of the ecdysone-triggered cascade both as a repressor (on the early 20E induced regulator genes) and an inducer of the β FTZ-F1 competence factor. By using DHR4 mutants it was suggested that DHR4 is involved in assessing critical weight and duration of larval development, linking 20E signaling to this process. It is possible that a similar molecular signaling is present in copepods and that the size of immature *L. salmonis *is the trigger for the onset of vitellogenesis. However, further studies are necessary to verify this hypothesis.

### Many novel genes in the *L. salmonis *genome

The initial analysis of individual *L. salmonis *EST sequences revealed that approximately 43% had no significant hits in GenBank using the nr-database. Even the re-annotation of the contigs identified by microarray analysis revealed a total of 41% novel proteins. Similar high proportion of novel genes was one of the interesting discoveries when the first non-mammalian genomes were sequenced. When the first metazoan animal (*C. elegans*) was completely sequenced the initial analysis revealed that more than 50% of the 19,000 genes were classified as novel [51]. Similar figures were seen in other "early" sequenced genomes. Obviously the most important reason for the high number of novel genes was that few organisms were completely sequenced and, hence, few "complete" dataset were available for comparison. This is illustrated in the worm book [52], where at present only 12.3% of the *C. elegans *protein coding genes are classified as "function unknown". Currently, no copepod genome has been sequenced and the closest sequenced relatives to the salmon louse are insects like *Drosophila *and *Anopheles*. This is obviously the main reason for the high number of novel proteins in the salmon louse but considering the increasing number of sequenced genomes, 40–50% novelty indicates that the copepods have a distinct proteome (in terms of the number of novel proteins) compared to terrestrial arthropods. This is further indicated by the fact that searching the  BaNG database [53] crustacean section with 35 novel proteins (see additional file 4) only resulted in six marginal (e-value 7E-6 to 3E-12) hits (data not shown). The large proportion of novel transcripts in *L. salmonis *was further demonstrated by the fact that of 4,586 validated nuclear *L.salmonis *ESTs 51% showed hits with insignificant values when compared to the only and recently available crustacean genome (*D. pulex*) [17].

Considering that reproduction processes have been intensively studied in many organisms, the number novel genes (38%) differentially transcribed during *L. salmonis *egg production (group 1 in the microarray study), was surprisingly high. The fact that conserved proteins in *L.salmonis *genes have the same low identity with homologous proteins in humans and the much closer related crustaceans and insects, further demonstrates the potential for discovery of unknown genes and "new" biological processes by sequencing genomes representing unstudied groups like copepods, instead of new species in already well studied groups.

## Conclusion

We have analyzed 7021 ESTs from the parasitic copepod *Lepeophtheirus salmonis*. Annotation demonstrated high level of novel genes (40%) and sequence identity analysis demonstrated that the identity of *Lepeophtheirus salmonis *proteins with homologous proteins in crustaceans and terrestrial arthropods is not higher than with homologous proteins in fish and humans. We have used 7 k microarrays to identify 120 genes, of which 40% are novel, involved in sexual maturation and egg production.

## Methods

### Animal material

Laboratory-reared strains of salmon louse were used. Infectious copepodids are produced in small buckets with flow-through water, starting with egg strings. The copepodids are allowed to infect naive Atlantic salmon kept in flow through water tanks. At relevant time points after infection, the salmon is anesthetized and salmon louse of interest collected and conserved for later use. In the present study adult fertilized individuals of *L. salmonis *were sampled during the period of maturation from pre-ad II to mature egg producing adults (app 14 days), photographed and stored on RNA later^® ^according to the manufactures recommendations. In the microarray experiment adult animals were divided into six different morphological groups (T1#150;T6) based on the cephalothorax/genital complex length (mm) ratio (see Figure 2) and the degree of genital filling/eggstring development. Five (in one case four) lice from each group were used as biological parallels in the microarray analyses. In addition 5 pre-ad II females were defined as T0. Pre-ad II males and adult males were sampled for comparison between the sexes.

### cDNA library construction and EST sequencing

Total RNA was extracted by Trizol (Sigma) and by RNeasy Mini Kit (Qiagen) followed by an enrichment of polyA RNA by Poly(A) Pure ™ or Poly(A) Purist ™ (Ambion) respectively. Two cDNA libraries (mRNA from female intestine and mRNA from the entire mature female) were constructed in lambda ZAP as described elsewhere [18]. In addition, two cDNA libraries (pre-ad II female and adult male) were constructed directly in the pBluscript SK+ vector as described by the manufactures (Stratagene). A subtracted cDNA library was constructed by using the PCR-select cDNA subtraction kit (Clontech). Mature adult female lice were used as the source for tester cDNA whereas adult males were used as driver cDNA. The lambda ZAP libraries were mass excised according to the manufactures instructions (Stratagene). All clones were blue-white screened and white clones were picked randomly from all the different libraries for plasmid purification. Bacteria were grown overnight in 96 well dishes (Millipore) and plasmids were purified according to the recommendations from the manufacturer (Millipore). Clones were sequenced using vector primers T3, m13f or m13f and BigDye chemistry (Applied Biosystems). The Vector NTI software package was used for sequence handling and analysis unless otherwise stated. After retrieval, the EST sequences were trimmed for vector sequence and quality assessment using options in the ContigExpress. Each EST was trimmed until there were less than 3 ambiguous bases per 25 bases. All approved ESTs were further analyzed by NCBI BlastX and BlastN searches in GenBank using the non-redundant database. In the primary annotation process a significant hit was defined as one with an Expectation value (*E*-value) less than 5.5 × 10^-5^. Based on this analysis the ESTs were annotated as similar to the sequence showing the highest score. In addition, we searched the Crustacea section in the BaNG database [53] with the 27 protein sequences used for similarity calculations and we compared 4,586 validated nuclear ESTs with the recently available *D. pulex *genome [17] using Blast. All ESTs were then clustered by the Contig Express module in the Vector NTI package using a minimum overlap of 50 bp, an identity of 0.9 and a cut off score of 20. Prior to the clustering, identified mitochondrial genes were removed. For *LsVit2, LsVit2 *and *LsVit-like*, full-length sequences were obtained by supplementing EST contigs with 5'RACE clones using SMART™RACE (Clonetech) and primer walking sequencing. Sequence data from this study have been submitted to GenBank and accession numbers are available in additional file 4.

### Microarray design and production

Probes were amplified from the individual cDNA clones by PCR using pBluescript-specific primers (TAATACGACTCACTATAGGGATAGGGCGAATTGGGTACCG and TAATACGACTCACTATAGGGAAAGGGAACAAAAGCTGGAGC). PCR reactions (100 μl) contained 10 μl 10 × reaction buffer (Promega), 160 μM MgCl_2_, 100 μM dNTPs, 0.15 μM of each primer and 2.5 U Taq U polymerase (Promega). An initial 2 min denaturation was followed by 30 PCR cycles (94°C for 30 sec, 60°C for 15 sec and 2 min elongation at 72°C) and a final 10 min elongation at 72°C. The PCR products were purified using Millipore Montage™ PCR_μ96 _Plate according to manufactures instructions. All purified probes were checked for size and purity by gel-electrophoresis (Invitrogen E-gel 96well 2% (GP). Furthermore, probe concentrations (70 ± 24 ng ng/μl) were estimated using Pico Green^® ^dsDNA quantitation reagent (Molecular Probes) according to the manufactures protocol and measured on a Fluorstar Optima (BMG Labtechnoologies). Probes (70–120 ng/μl) in 50% DMSO were printed on Aminosilane coated slides (Corning^® ^UltraGaps™) at 20–22°C and 45–55% relative humidity using a BioRobotics, Micro Grid II arrayer (Genomic Solutions^®^) with Mikrospot 10 K split pins. Slides were dried in a desiccator cabinet for 24–48 hours and DNA crosslinked at 350 mJ/cm2 using a UV-Stratalinker 2400, (Stratagene Inc.). The 7776 spots were printed in 24 subarrays, 7008 *L. salmonis *probes, 72 (triplicate on each subarray) *L. salmonis *reference gene *EF1α *[54], 360 negative controls mouse ESTs (15 on each subarray) and the rest were "no probe spots".

### RNA isolation, cDNA labeling for microarray hybridization

RNA was isolated for individual animals using the RNAeasy Mini kit (Qiagen) according to the manufactures recommendations. The RNA was DNAse treated by TURBO DNA-free™ (Ambion) according to the supplied protocol and Superase (Ambion) was added. The RNA samples were frozen at -80°C until analysis. One aliquot was used for RNA integrity and quantity measures using the Agilent 2100 Bioanalyzer and NanoDrop Spectrophotometer (OD 260/280 and 260/230 ratios), respectively. Another aliquot was used for cDNA synthesis and labeling using Fair Play^® ^Microarray Labeling Kit (Stratagene) according to the manufactures instructions. Typically, 10 ug total RNA was used for cDNA generation. In a few cases less RNA (down to 5 μg) were used due to lower RNA yields, typically from the smaller single pre-adult lice. Samples (Cy5 labeled) where hybridized against a common standard (Cy3 labeled) reverse transcribed and labeled in parallel. The reference RNA was a pooled and aliquoted mRNAs mix from adult females, pre-adult females, adult males and pre-adult males (1:1:1:1), tested and used as described for the samples. Labeling efficiency and quantity of labeled cDNA was determined using the NanoDrop Spectrophotometer and identically quantity of sample and standard was used in all hybridizations. Slides were pre-hybridized in 20 × SSC, 10% SDS and 1%BSA for about 45 min at 65°C followed by washing twice in water and once in isopropanol. Slides were dried by centrifugation in a mini-centrifuge. Sample and reference was unified, diluted in Tris buffer pH 8.0 and centrifuged using microcon YM-30 columns (Millipore). After sample denaturation (100°C, 2 min) hybridization was performed at 60°C over night with rotation using Agilent 2 × hybridization buffer (250 μl) in Agilent hybridization chambers. The slides were put in 2 × SSC/0.1%SDS at 65°C to remove gasket slide and then washed for 5 min in 1 × SSC at 65°C, for 5 min in 0.2 × SSC at RT, for 45 sec in 0.05 × SSC at RT, and spinned dry (mini-centrifuge). Dye swap quality control experiments were performed with two samples and technical replicates performed with five samples.

### Microarray analysis

Slides were scanned directly after the washing procedure using an Agilent scanner at a resolution of 10 um with default settings. The scanned microarray images were analyzed using the GenePix Pro 6.0 software package and exported as image quantization files (gpr- and jpg-files). Each gpr- and jpg-file was then further processed in the J-Express (version 2.7) software package [55] and organized into a gene expression matrix where each row represents a clone, each column represents a sample (salmon louse) and each cell contains a log_2 _(sample vs. reference) ratio. The processing of image quantization data was performed by removing (1) all "empty" and "control" spots (696 spots) and all spots flagged by the GenePix software package or manually as "not found" or "bad" and (2) all spots with a signal to noise ratio (reported by GenePix) below 3.0 in both channels before a global lowess normalization [56] was applied to all remaining probes.

To prepare the gene expression matrix for further downstream analysis, we applied a KNN (k = 10) imputation to make sure an expression value existed for all genes in all samples [57]. Prior to imputation, we removed all genes having more than 80% missing values, giving an expression matrix with 6653 clones. To remove ESTs not in the scope of this project, we used a self-organizing map to group ESTs according to expression profiles. All time courses of red/green ratios for all ESTs were sorted via self-organizing maps with Euclidian distance [21,22] (25 cluster (5 × 5) neuron map). The self-organizing map shows clusters of ESTs sorted by cluster variance. To identify candidate gene clusters, which are most regulated over time the clusters with highest within cluster variance were selected. Using a trimmed group mean of samples from the same development stage a combined dataset was created. To further explore gene expression similarities corresponding to development stage, we applied a correspondence analysis to the complete dataset and to a group-combined expression matrix (samples within a development group was combined using a trimmed mean) [23]. Developmental group membership was also explored by self-organizing map. Finally, probe quality of all spots from the chosen clusters was checked. All spots were the probe agarose gel electrophoresis test showed double band, smear or size below 400 bp was removed. Secondly, spots were filtered using cut off values for average signal to noise ratio (SNR 635, 532), spot intensity (Mean F635, F532) for both red and green channel, and for the percentage of feature pixels with intensities more than two standard deviations above the background intensity (at wavelength 635 or 532 (% > B635+2SD)). The cut-off values were set based on evaluation of all probes in contigs with more than 20 ESTs, setting the cut of at the level where individual probes gave inconsistent results compared to other probes representing the same contig. These cut off were: SNR< 40, F< 100 and > 10000, B+2SD< 60.

The consensus sequences of differentially expressed genes, edited contigs and re-sequenced singletons were checked for ORFs and re-annotated using BlastP (if ORF) and BlastX.

We provide MIAME-compliant description of the microarray study, available in the arrayexpress database [58], accession number E-BASE-5.

### Northern blots

Total RNA from pre-adII, T1, T3, T5 (female) and pre-adII and adult male lice (3.2 μg) was mixed with Northern Max Formaldehyde Loading Dye (Ambion), denaturated (10 min, 80°C), ethidium bromide added and samples run on a 1% denaturizing agarose gel (MOPS). Quality and quantity of RNA was evaluated under UV light before RNA was blotted onto Hybond-N nylon membrane (Amersham) using standard upward blotting technique in 10 × SSC blotting buffer and crosslinked at 120 mJ/cm2 using a UV stratalinker (Stratagene). PCR probes were produced as previously described for microarray probes and analyzed on agarose gel. The individual probes were cut out of the gel and purified using DNA Gelextraction (Millipore). PCR product (25 ng) was labeled with ^32^P 6000 Ci/mmol (GE Healthcare) using StripEZ^®^DNA (Ambion) and cleaned with Quiaquick Nucleotide Removal Kit (Qiagen) according to manufactures instructions. After denaturation (90°C, 10 min) individual probe were hybridized to individual membranes at 68°C over night (Perfect Hyb ^4M ^Plus hybridization buffer (Sigma), 5 ml per filter). The membranes were washed with 2 × SSC/0.1% SDS (2 × 5 min RT), 1 × SSC/0.1% SDS (1 × 15 min RT), 0.5 × SSC/0.1% SDS (2 × 10 min 68°C) followed by exposure on Kodak BioMax MS for 1 to 3 days. Membranes were stripped once (for re-use) with StripEZ^®^DNA (Ambion) according to manufactures instructions.

## Authors' contributions

CE produced all probes for microarray-production, performed all microarray experiments, performed microarray data analysis and contributed to the manuscript. PF planned the study, supervised the microarray production and microarray experiments and wrote the paper together with FN. BD contributed to the microarray experimental design and performed microarray data analyses. IJ supervised the microarray design and the microarray data analysis, and contributed to the manuscript. BK contributed to microarray design and printed the microarrays. FN planned and supervised the study, performed all EST analyses and wrote the paper together with PF. All authors read and approved the manuscript.

## Supplementary Material

Additional file 1 Identification of groups of genes (clusters) with similar expression profiles using a 5 × 5 self-organizing map (SOM). Highest up-regulated profiles are up to the left while max down-regulated can be seen down to the left. Click here for file

Additional file 2 Correspondence analysis (developmental stage and transcription) including all samples. Samples are located in adjacency with other samples from the same developmental group or in the vicinity of a neighboring time point in development. Click here for file

Additional file 3 Self-organizing map (SOM). For each sample, the SOM creates 10 × 10 cells where each cell corresponds to the mean expression value for one neuron in the map. Similar color pattern can be seen for biological replicates (samples within each development group). Click here for file

Additional file 4 List of accession numbers and characteristics of all described *L.salmonis *gene contigs including hyperlinks to GenBank. Click here for file

Additional file 5 Validation of microarray results by comparing microarray transcription profiles with Northern blots for 7 different *L.salmonis *contigs. Click here for file
