# Supplementary material for: Salmon louse (Lepeophtheirus salmonis) transcriptomes during post molting maturation and egg production, revealed using EST-sequencing and microarray analysis
Source: BMC Genomics. 2008 Mar 10;9:126. doi: 10.1186/1471-2164-9-126 (PMC2329643; doi:10.1186/1471-2164-9-126)
Supplement: Additional file 1 — Identification of groups of genes (clusters) with similar expression profiles using a 5 × 5 self-organizing map (SOM). Highest up-regulated profiles are up to the left while max down-regulated can be seen down to the left. [file 1471-2164-9-126-S1.pdf]

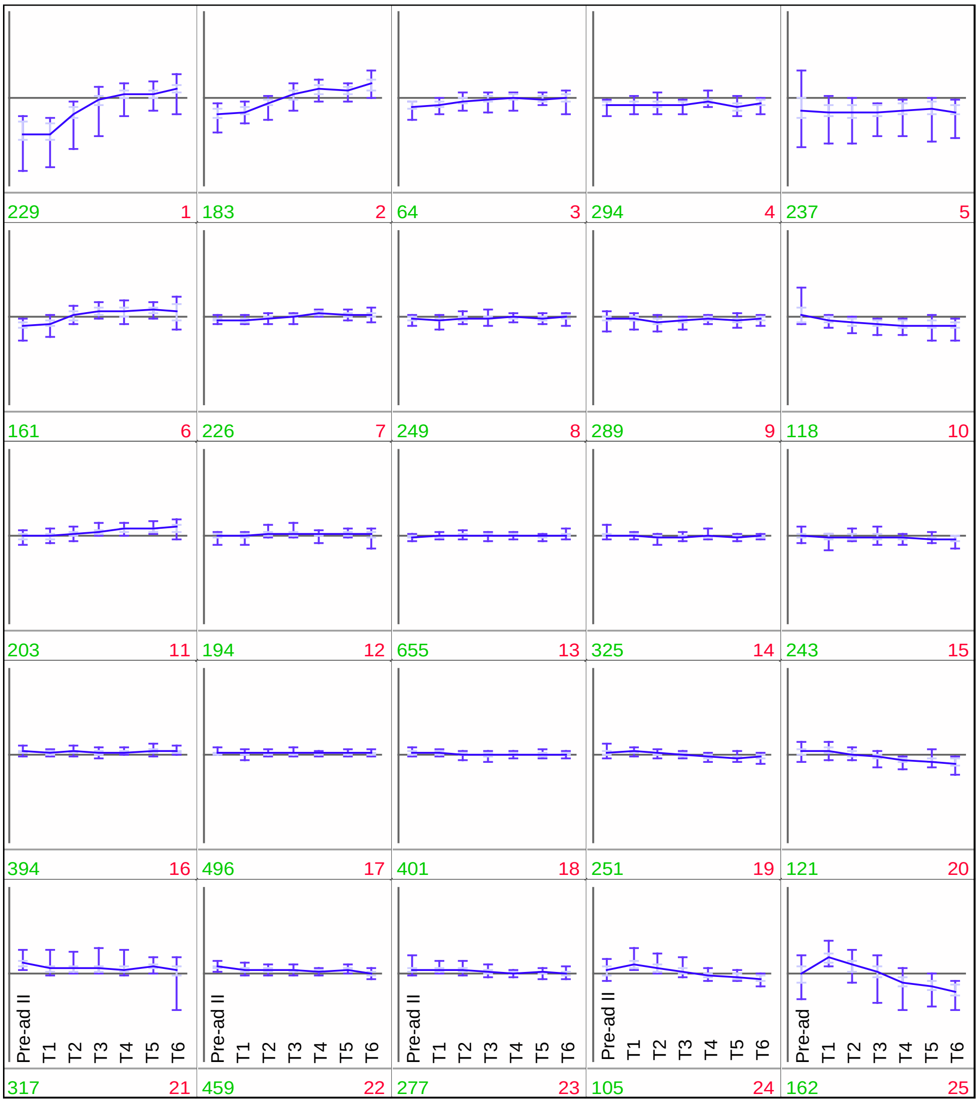

Additional file 1: Grouping of expression profiles using a 5x5 self-organizing map (SOM). Interesting profiles were found in cluster 1, 2, 6, 24 and 25. The first 3 clusters show an up-regulation from T2 while cluster 24 and 25 are upregulated at T1 and subsequently downregulated. Dark blue colors corresponds to the mean expression profile for each cluster, dark blue bars corresponds to cluster standard deviation, light blue color bars represents cluster max-min values, the green number represents cluster size and the red number represents cluster ID.
