# Supplementary material for: Salmon louse (Lepeophtheirus salmonis) transcriptomes during post molting maturation and egg production, revealed using EST-sequencing and microarray analysis
Source: BMC Genomics. 2008 Mar 10;9:126. doi: 10.1186/1471-2164-9-126 (PMC2329643; doi:10.1186/1471-2164-9-126)
Supplement: Additional file 3 — Self-organizing map (SOM). For each sample, the SOM creates 10 × 10 cells where each cell corresponds to the mean expression value for one neuron in the map. Similar color pattern can be seen for biological replicates (samples within each development group). [file 1471-2164-9-126-S3.pdf]

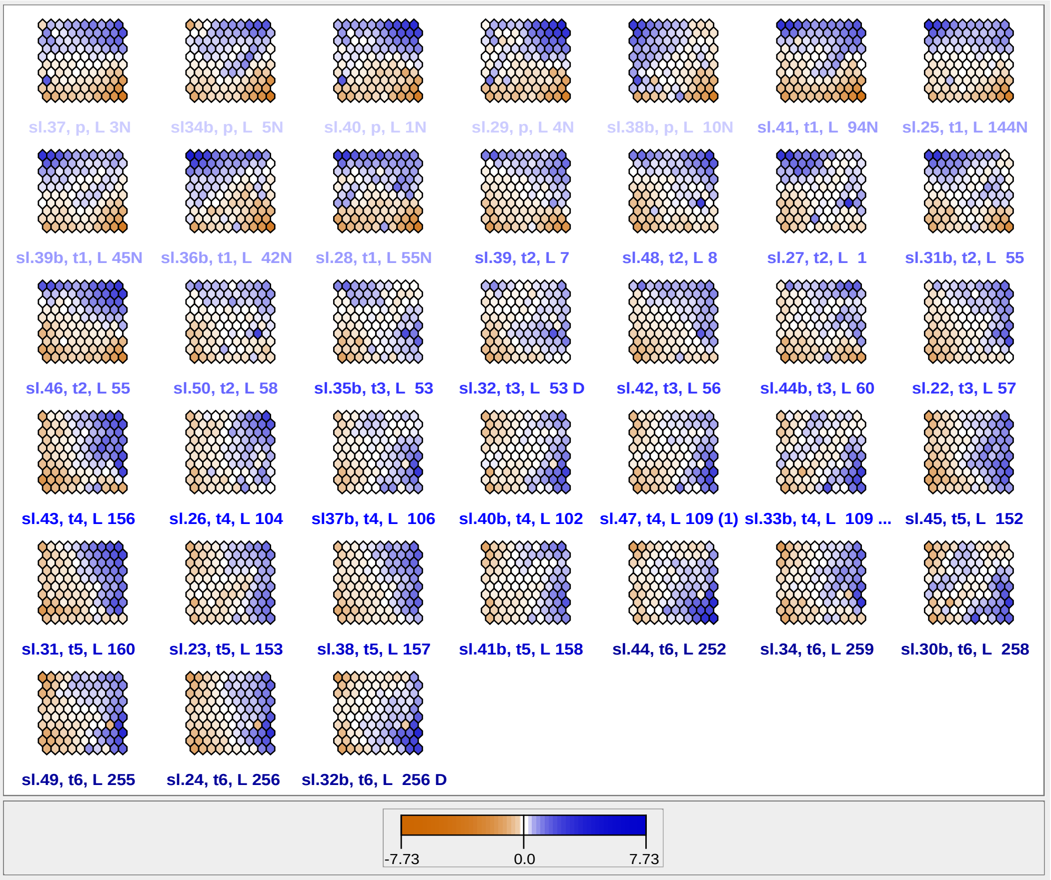

Additional file 3: Self-organizing map (10x10 neuron mean heat map). For each sample, the SOM creates 10x10 cells where each cell corresponds to the mean expression value for one neuron in the map. Similarity in color pattern can be confirmed for most samples within each development group. Some outliers exist, but patterns in these often resemble patterns from groups adjacent in the time series (e.g the pattern for slide 46, t2 looks more like a t1 sample (see patterns for slide 41 and 25) which agrees with the full CA analysis, se additional file 2).
