# Supplementary material for: Salmon louse (Lepeophtheirus salmonis) transcriptomes during post molting maturation and egg production, revealed using EST-sequencing and microarray analysis
Source: BMC Genomics. 2008 Mar 10;9:126. doi: 10.1186/1471-2164-9-126 (PMC2329643; doi:10.1186/1471-2164-9-126)
Supplement: Additional file 5 — Validation of microarray results by comparing microarray transcription profiles with Northern blots for 7 different L.salmonis contigs. [file 1471-2164-9-126-S5.pdf]

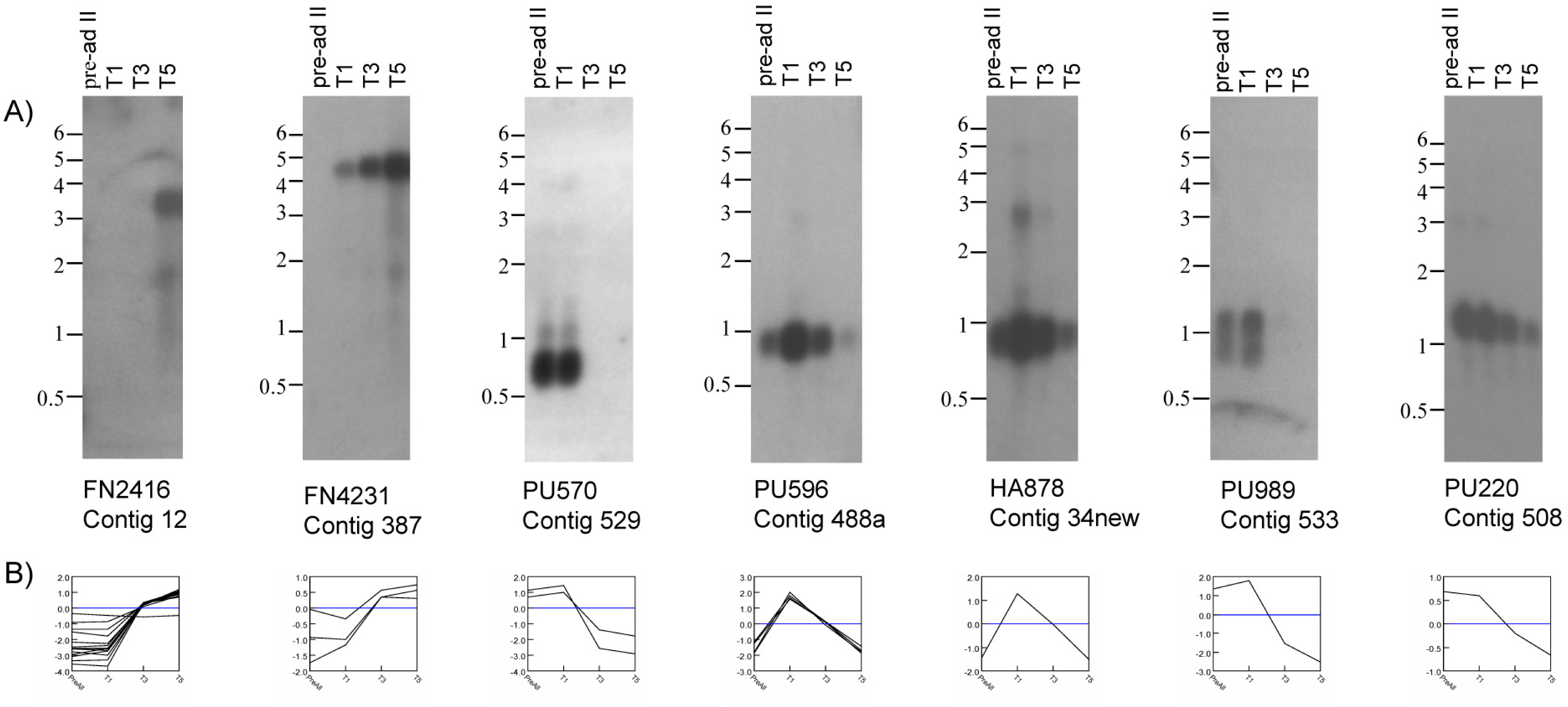

Additional file 5. A) Northern blot analysis of two genes in Group 1 (Contig12, Contig387) and five genes in Group 2 (Contig529, Contig488a, Contig34new , Contig533, Contig508). B). Microarry data from same samples (pre-ad II, T1, T3, T5)
