# Supplementary material for: Salmon louse (Lepeophtheirus salmonis) transcriptomes during post molting maturation and egg production, revealed using EST-sequencing and microarray analysis
Source: BMC Genomics. 2008 Mar 10;9:126. doi: 10.1186/1471-2164-9-126 (PMC2329643; doi:10.1186/1471-2164-9-126)
Supplement: Additional file 2 — Correspondence analysis (developmental stage and transcription) including all samples. Samples are located in adjacency with other samples from the same developmental group or in the vicinity of a neighboring time point in development. [file 1471-2164-9-126-S2.pdf]

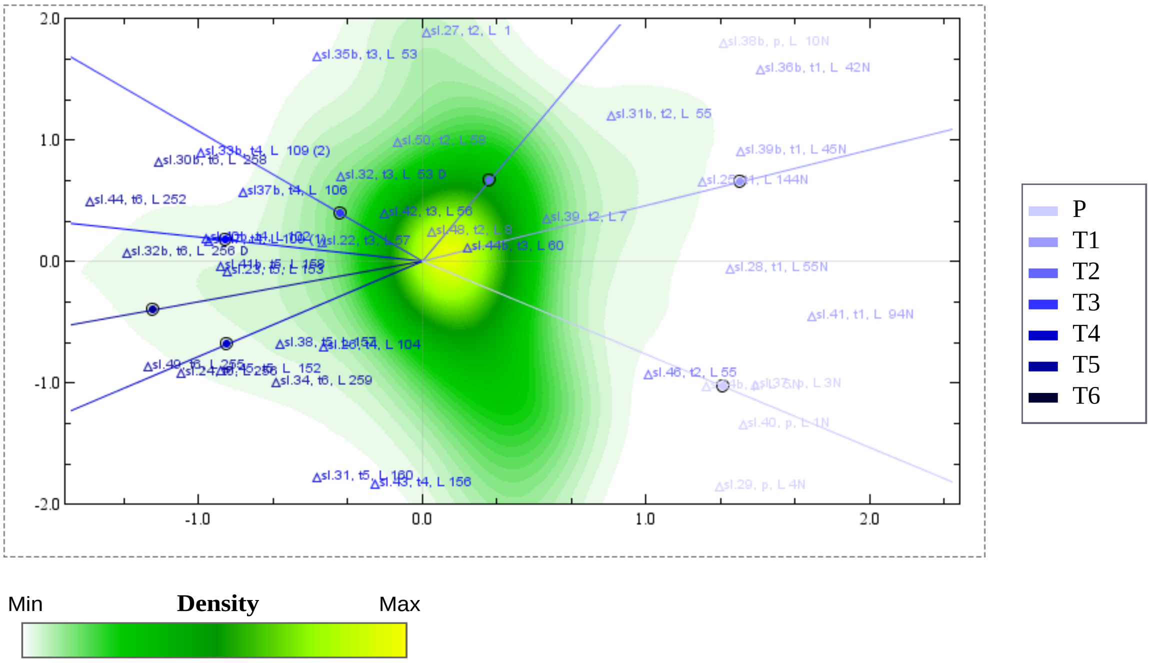

Additional file 2: Correspondence analysis including all samples. Samples have colour representing each development group (colour codes are provided to the right). A vector showing the mean of each development group is also added. The green/yellow color in the background indicates gene density.
